# Biosensor-guided rapid screening for improved recombinant protein secretion in *Pichia pastoris*

**DOI:** 10.1186/s12934-023-02089-z

**Published:** 2023-05-03

**Authors:** Laura Navone, Kaylee Moffitt, James Behrendorff, Pawel Sadowski, Carol Hartley, Robert Speight

**Affiliations:** 1 School of Biology and Environmental Science, Faculty of Science, Queensland University of Technology (QUT), Brisbane, QLD 4000 Australia; 2 ARC Centre of Excellence in Synthetic Biology, Queensland University of Technology (QUT), Brisbane, QLD 4000 Australia; 3 Central Analytical Research Facility (CARF), Queensland University of Technology (QUT), Brisbane, QLD 4000 Australia; 4 CSIRO Environment, Canberra, ACT 2600 Australia

**Keywords:** Protein production, Screening, Biosensor, Split GFP, Yeast

## Abstract

*Pichia pastoris (Komagataella phaffii)* is widely used for industrial production of heterologous proteins due to high secretory capabilities but selection of highly productive engineered strains remains a limiting step. Despite availability of a comprehensive molecular toolbox for construct design and gene integration, there is high clonal variability among transformants due to frequent multi-copy and off-target random integration. Therefore, functional screening of several hundreds of transformant clones is essential to identify the best protein production strains. Screening methods are commonly based on deep-well plate cultures with analysis by immunoblotting or enzyme activity assays of post-induction samples, and each heterologous protein produced may require development of bespoke assays with multiple sample processing steps. In this work, we developed a generic system based on a *P. pastoris* strain that uses a protein-based biosensor to identify highly productive protein secretion clones from a heterogeneous set of transformants. The biosensor uses a split green fluorescent protein where the large GFP fragment (GFP1-10) is fused to a sequence-specific protease from Tobacco Etch Virus (TEV) and is targeted to the endoplasmic reticulum. Recombinant proteins targeted for secretion are tagged with the small fragment of the split GFP (GFP11). Recombinant protein production can be measured by monitoring GFP fluorescence, which is dependent on interaction between the large and small GFP fragments. The reconstituted GFP is cleaved from the target protein by TEV protease, allowing for secretion of the untagged protein of interest and intracellular retention of the mature GFP. We demonstrate this technology with four recombinant proteins (phytase, laccase, β-casein and β-lactoglobulin) and show that the biosensor directly reports protein production levels that correlate with traditional assays. Our results confirm that the split GFP biosensor can be used for facile, generic, and rapid screening of *P. pastoris* clones to identify those with the highest production levels.

**Supplementary Information:**

The online version contains supplementary material available at 10.1186/s12934-023-02089-z.

## Introduction

The methylotrophic yeast *P. pastoris* is a popular expression platform for the production of heterologous proteins of biopharmaceutical or industrial interest. *P. pastoris* is a GRAS (generally regarded as safe) strain that grows to high cell densities in bioreactors and has high capacity for secreting heterologous proteins [1]. Recombinant proteins produced commercially using this yeast include biopharmaceutical proteins (e.g., Kalbitor®, Jetrea®, human insulin and analogues) and industrial enzymes (e.g., phytases, trypsin, nitrate reductase, phospholipase C, collagen, proteinase K), and there is a growing interest in using *P. pastoris* as a production platform for food proteins [1, 2]. As a eukaryote, it is also capable of post-translational modifications like disulfide bond formation and glycosylation. This yeast is easy to manipulate and culture and it naturally secretes low amounts of endogenous proteins which simplifies product purification at industrial scale [1, 3]. These advantages means that production of recombinant proteins in *P. pastoris* can be easier and less expensive than other eukaryotic expression systems like insect and mammalian cells systems.

Recent advances in synthetic biology have greatly extended the toolbox for genetic engineering of *P. pastoris*. Molecular tools include synthetic promoters for fine-tuning of expression, glyco-engineered strains to mimic human glycol-forms for biopharmaceuticals, CRISPR/Cas9 technology for genomic engineering and several methods for the knockout of multiple genes and over-expression of entire pathways [4–8]. High-quality genome sequences and metabolic models have also contributed to a more comprehensive understanding of *P. pastoris* and allowed for rational strain engineering [1].

Despite major developments of *P. pastoris* as an expression system, achieving sufficiently high specific productivities of secreted proteins to be economically viable at commercial scale is still a challenge. Fermentation titers are an important determinant of the cost of recombinant protein manufacturing. Selection of *P. pastoris* clones that maximise production is critical for bioprocess development, and hundreds to thousands of clones may need to be screened to select for good producers due to high clonal variability between transformants.

For generating *P. pastoris* expression strains, cells are transformed with linear integrative expression cassettes that target a specific position in the genome, such as the alcohol oxidase 1 (AOX1) locus. A common strategy to select for highly productive strains is to screen for clones having multi-copy integrations of the desired expression cassette. The majority of the transformants will have a single copy of the expression cassette and a high number of clones must be screened to select high copy number strains (about 1% of the transformants are multiple copy clones) [9]. Furthermore, single or multiple copy integrations can randomly occur in other regions of the genome which increases clonal variability. Screening for multicopy integrants can be a strategy for selecting clones with higher expression, but copy number does not always have a direct correlation with protein production [9, 10].

A direct assay of the recombinant protein of interest is preferable for screening of clones but developing suitable high throughput screening methods for protein secretion can be time consuming and technically challenging. Semi high-throughput small-scale cultivation is possible using deep-well plates, but selection of clones still relies on specific enzymatic assays or immunoblotting that both require some level of protein extraction and processing. A straightforward method that can be generally applied to any protein would be a very valuable tool to facilitate selection of the best producers. Here, we developed a split-GFP protein biosensor that allows rapid, high-throughput and direct measurement of recombinant protein production prior to sample collection. This method can be applied to any heterologous protein produced in *P. pastoris*.

The biosensor is based on soluble self-associating fragments of superfolder GFP developed to tag and detect soluble and insoluble proteins in vivo [11]. The large fragment GFP1-10 (23.7 kDa) is fused to tomato etch virus (TEV) protease and expressed constitutively in the ER, while the small GFP11 fragment (1.8 kDa) is used to tag the recombinant protein at the C-terminal next to a TEV cleavage site [12]. Folding of the complete GFP1-11 in the endoplasmic reticulum of the cell allows relative amounts of recombinant protein passing through the secretory pathway to be determined.

Secretory proteins are synthesised and transported to the lumen of the ER for folding and processing [13]. A hydrophobic amino-terminal extension, the signal sequence, is responsible for targeting and translocation of proteins into the ER. Post-translational modifications like disulfide bond formation and glycosylation occurs in the lumen of the ER [13]. Once the protein has attained its folded and mature conformation it is transported to the Golgi for further processing and ultimately to the extracellular membrane for secretion [13]. The GFP11 tagged recombinant protein enters the ER where it complements the GFP1-10_TEV protease fusion increasing cell fluorescence. Total fluorescence directly correlates to amount of recombinant protein transiting through the ER. The presence of TEV protease allows for cleavage of the recombinant protein that is fully secreted to the extracellular media [12].

The split-GFP biosensor was tested for selection of clones producing two recombinant enzymes (phytase and laccase) and two structural milk proteins (β-casein and β-lactoglobulin). The increase of intracellular fluorescence over time was shown to be directly correlated to the amount of secreted recombinant protein. The biosensor is a rapid and facile tool that can be used to identify high productivity *P. pastoris* production strains for enzymes and structural proteins.

## Results

### Biosensor design using split-GFP fused to TEV protease

**Fig. 1:**
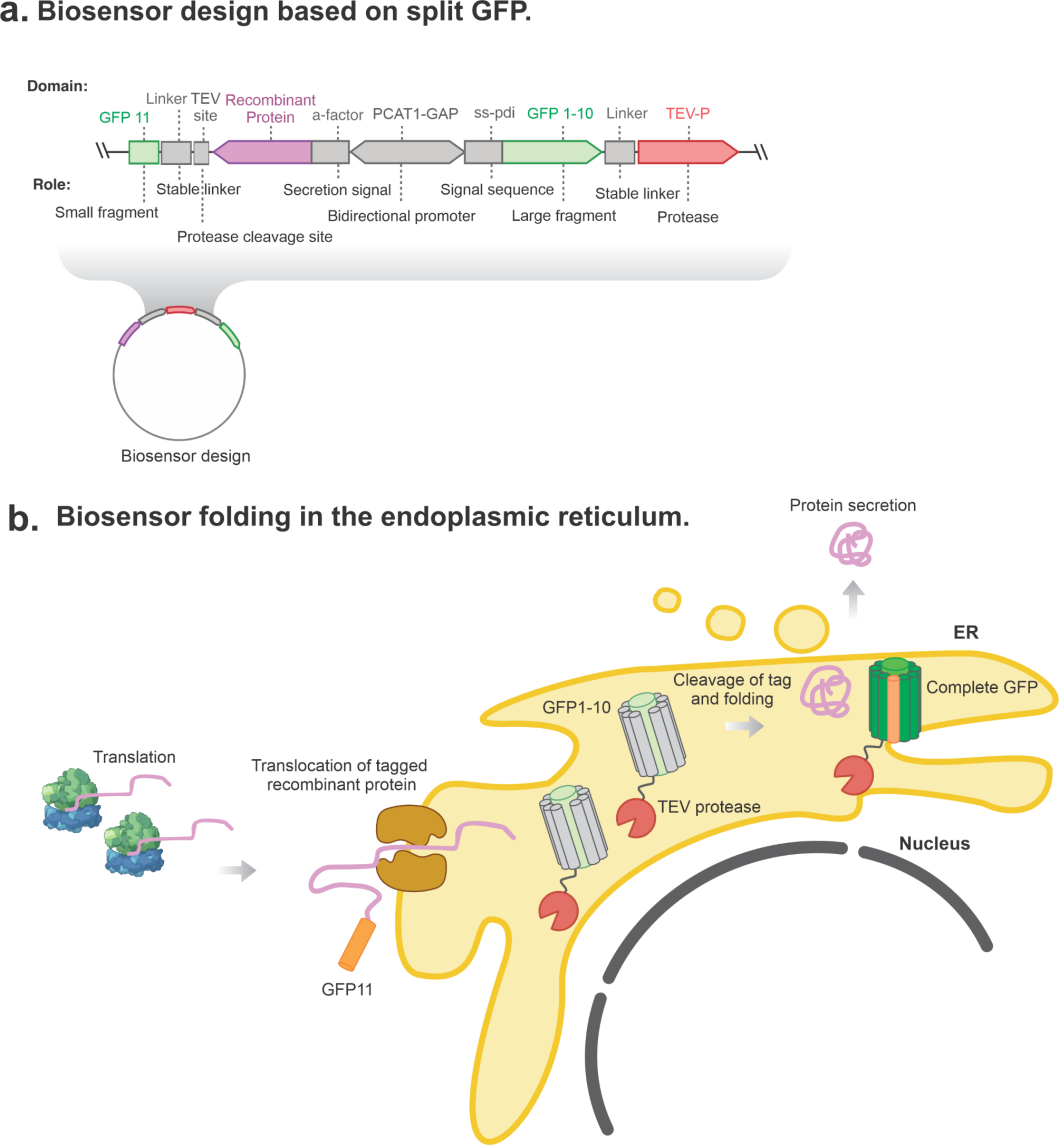
**Biosensor design and folding in the endoplasmic reticulum.** Schematics of the genetic design and folding of biosensor fragments upon recombinant protein production. **a**, Genetic design of biosensor fragment targeted to the endoplasmic reticulum and tagged recombinant protein with secretion signal. **b**, Complete biosensor folding in the endoplasmic reticulum and mechanism of fluorescence increase by accumulation of recombinant protein

We selected a previously-optimised split-GFP system for the biosensor design [11]. The large GFP fragment (GFP1-10, 23.7 kDA) was fused to a TEV protease and expressed constitutively under *P*_*GAP*_ in the ER of *P. pastoris* (Fig. 1). For this, the signal peptide from Pdi protein (protein disulfide isomerase) was added to the N-terminal sequence of GFP1-10_TEV to target to the ER. Furthermore, a HDEL sequence was incorporated at the C-terminal of GFP1-10_TEV to retain the fusion protein in the lumen of the ER and avoid secretion (Table 1).

The small GFP11 fragment (1.8 kDa) was fused to the C-terminus of recombinant protein genes, *Escherichia coli* phytase (AppA), *Trametes hirsuta* laccase 1 (Thi_lac1), bovine β-casein and bovine β-lactoglobulin. The genetic design included a TEV cleavage site for liberation of the GFP11 tag and recombinant protein secretion. We used the α-factor secretion signal and de-repressed methanol inducible promoter for expression of the recombinant protein fusions. *P*_*CAT1*_ is de-repressed under low concentrations of glucose and further induced by addition on methanol [7].

### Expression of recombinant enzymes and complete GFP folding in the endoplasmic reticulum

To test the functionality of our biosensor for screening *P. pastoris* protein producers, we first selected two recombinant enzymes to express, AppA phytase and Thi_lac1 laccase [14–16]. Both enzymes are of great industrial interest. Phytases are ubiquitously used as dietary supplement for swine and poultry to increase digestibility of phytate (myoinositol hexaphosphate), the main form of phosphorous storage in grains. Laccases catalyse the radical-mediated oxidation of substituted phenols using molecular oxygen as the final electron acceptor and are of broad interest for their catalytic promiscuity and potential for use in applications such as dye decolourisation and antibiotic degradation in wastewater treatment [14, 16]. While AppA phytase was previously expressed in *P. pastoris* with good productivity, to our knowledge Thi_lac1 laccase has not previously been produced heterologously in yeast [15, 17].

**Fig. 2:**
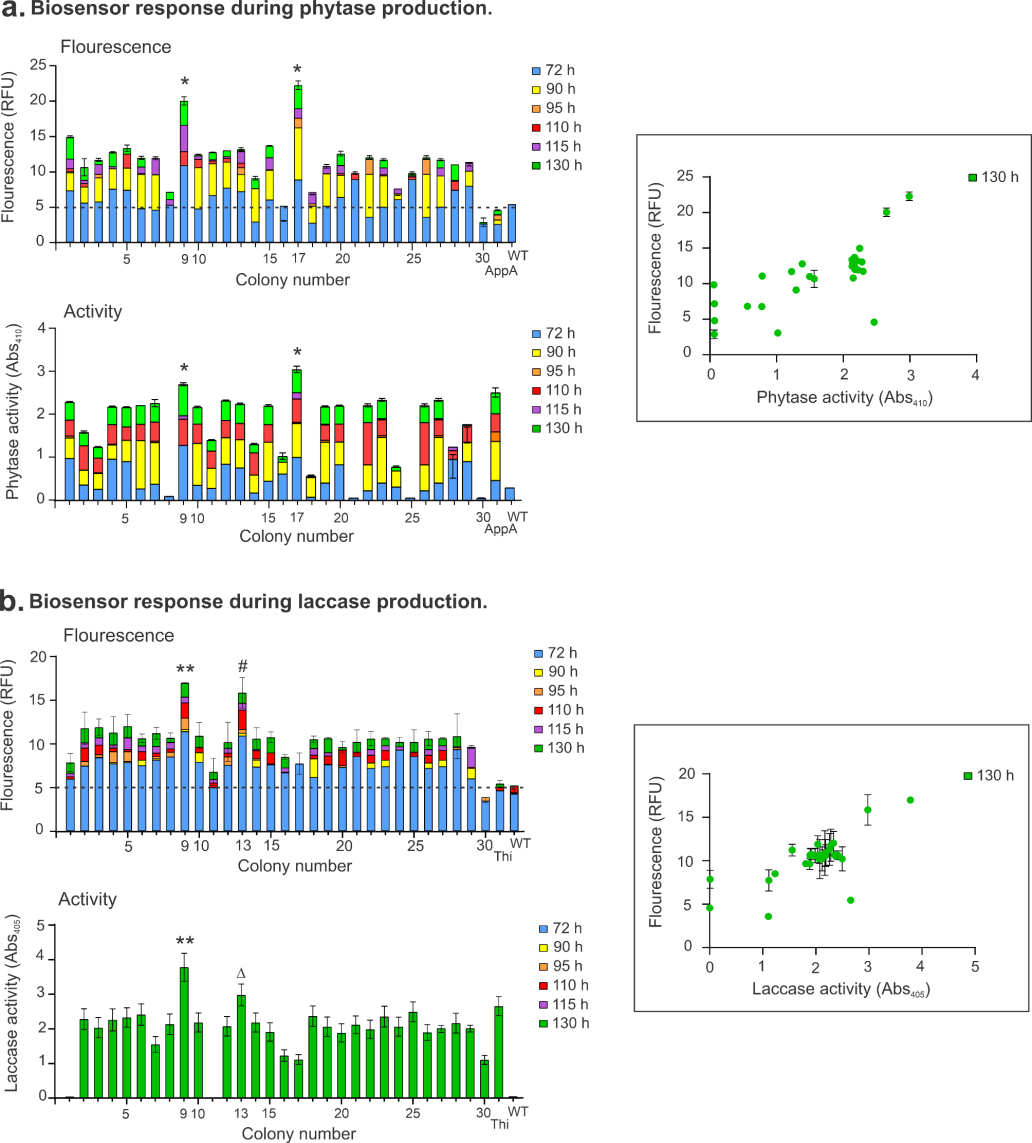
**Biosensor response during phytase and laccase production. a**, Phytase and **b**, laccase production under methanol induction at 72, 90, 95, 110, 115 and 130 h. Fluorescence is indicated as relative fluorescence units (RFU) and phytase and laccase activity as absorbance at 405 and 410 nm, respectively. Correlation between secreted enzyme activity and intracellular GFP fluorescence shown in framed graphs. Data are shown as the mean and standard deviation of three biological replicates and analysed using one-way ANOVA with multiple comparisons. The dashed line indicates background fluorescence at 485–520 nm in wild-type fermentations. *Significantly different to all colonies (p < 0.01). **Significantly different to all colonies except colony 13 at 130 h (p < 0.01). ^#^ Significantly different all colonies except colonies 2, 3, 4, 5 and 7 (p < 0.01). ^Δ^ Significantly different all colonies except colonies 2, 3, 4, 5,10 14, 18, 23, 25 and 28 (p < 0.01)

We transformed *P. pastoris* with biosensor DNA cassettes to express the recombinant tagged proteins and screened thirty to sixty transformants in each case. Fluorescence intensity at 485–520 nm was measured over time (Fig. 2). The increase in fluorescence corresponded to folding of complete GFP while recombinant protein was transported through the ER, the cleavage of the GFP11 tag from the recombinant protein by TEV protease enabled secretion of the enzymes to the extracellular media (Fig. 1).

Cells were grown in 96-deep-well plates in defined media supplemented with glucose and 1% methanol was added at 65, 72, 90, 95, 110, 115 and 130 h. Fluorescence was measured immediately before each methanol addition and 5 h afterwards. Results showed low fluorescence at 72 h which is expected from using *P*_*CAT1*_ de-repressed promoter to control the expression of recombinant proteins and by the addition of methanol at 65 h [18]. Fluorescence doubled for most transformants during methanol induction up to 130 h corresponding to increases of recombinant protein production. Dotted lines in Fig. 2 indicate background fluorescence of cells compared to the wild type MutS strain. We also conducted enzymatic activity tests for phytase or laccase on the supernatant from each transformant and correlated these results to fluorescence at 485–520 nm.

The greatest GFP fluorescence was measured in phytase-producing clones 9 and 17, and laccase-producing clones 9 and 13. These clones also secreted the greatest amount of functional phytase and laccase enzymes, as determined by activity assays using culture supernatants (Fig. 2).

The correlation between secreted enzyme activity and intracellular fluorescence intensity confirms that the biosensor responds to recombinant protein production. Overall, the genetic variability of the *P. pastoris* transformants is reflected in fluorescence intensity and the biosensor can be used for screening of protein producers.

To test if the expression of the split biosensor GFP1-10 under constitutive promoter affected cell growth, we determined the optical density at 600 nm for each transformant. Results showed that growth was not affected in any of the transformants in comparison to the wild type MutS strain (Additional file 1 Fig. S1). Only one transformant during AppA phytase screening presented a reduced growth rate which could indicate increased AppA phytase production after biomass normalisation (Additional file 1 Fig. S1). Overall, these results indicated that the expression of the GFP1-10_TEV protease fusion constitutively under *P*_*GAP*_ does not affect cell growth.

**Fig. 3:**
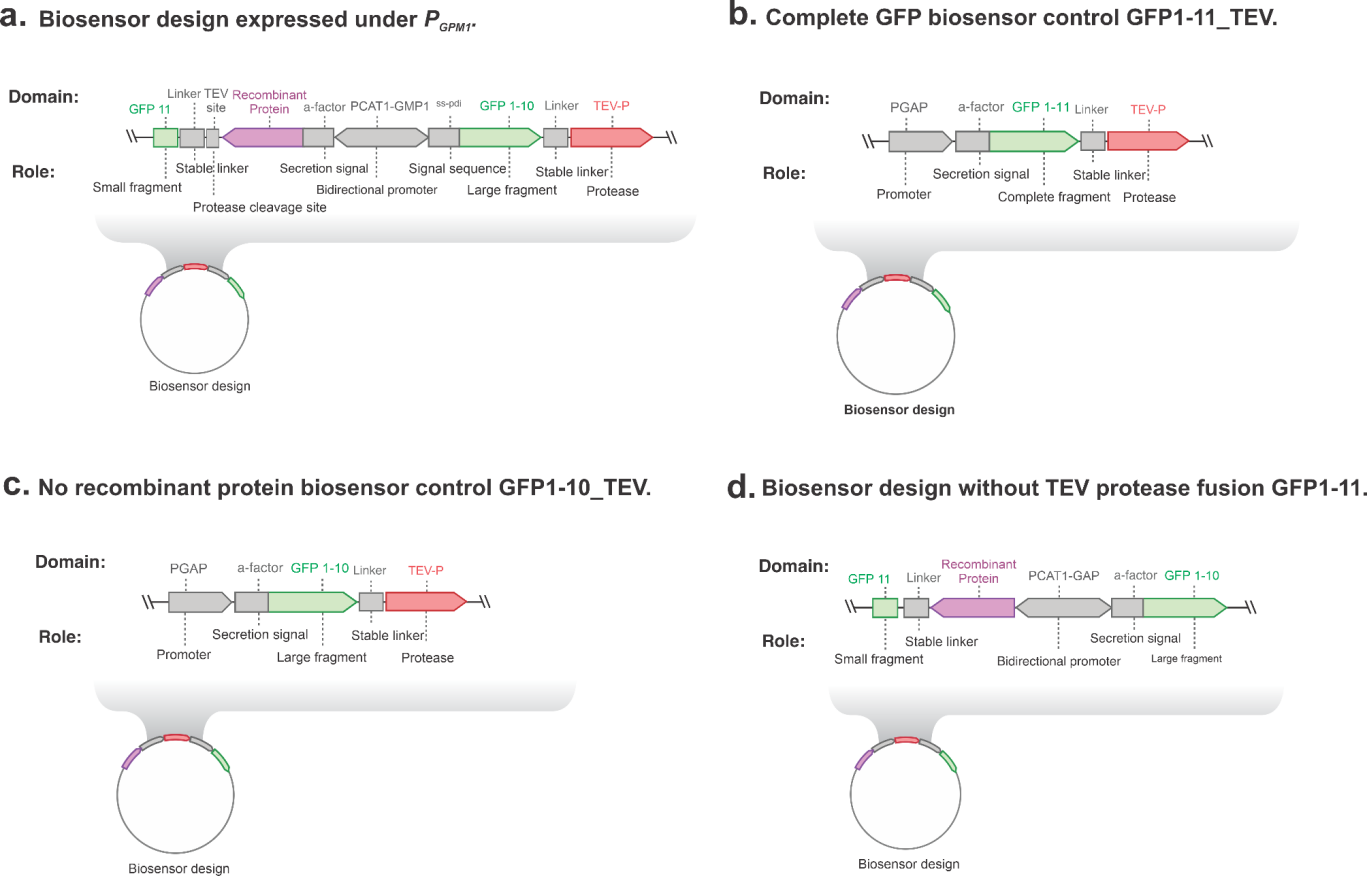
**Biosensor design controls and expression under*P***_***GPM1***_. Schematics of the genetic designs **a**, Genetic design of biosensor expressed under ***P***_***GPM1***_. **b**, Genetic design of complete GFP biosensor (GFP1-11_TEV). **c**, Genetic design of biosensor control without expression of recombinant protein (GFP1-10_TEV). **d**, Genetic design of biosensor without TEV protease fusion (GFP1-11).

To evaluate potential hindering of recombinant protein production due to the use of the strong *P*_*GAP*_ for expression of the biosensor fusion, we also constructed a design using the *P*_*GPM1*_ constitutive promoter which is weaker than *P*_*GAP*_ (Fig. 3a). Other biosensor controls were also constructed to evaluate correct folding of GFP fragments and fluorescence response (Fig. 3c-d). Designs included complete GFP fused to TEV protease (GFP1-11_TEV) without an additional recombinant protein (Fig. 3b) and GFP1-10 fused to TEV protease without a recombinant protein (Fig. 3c).

**Fig. 4:**
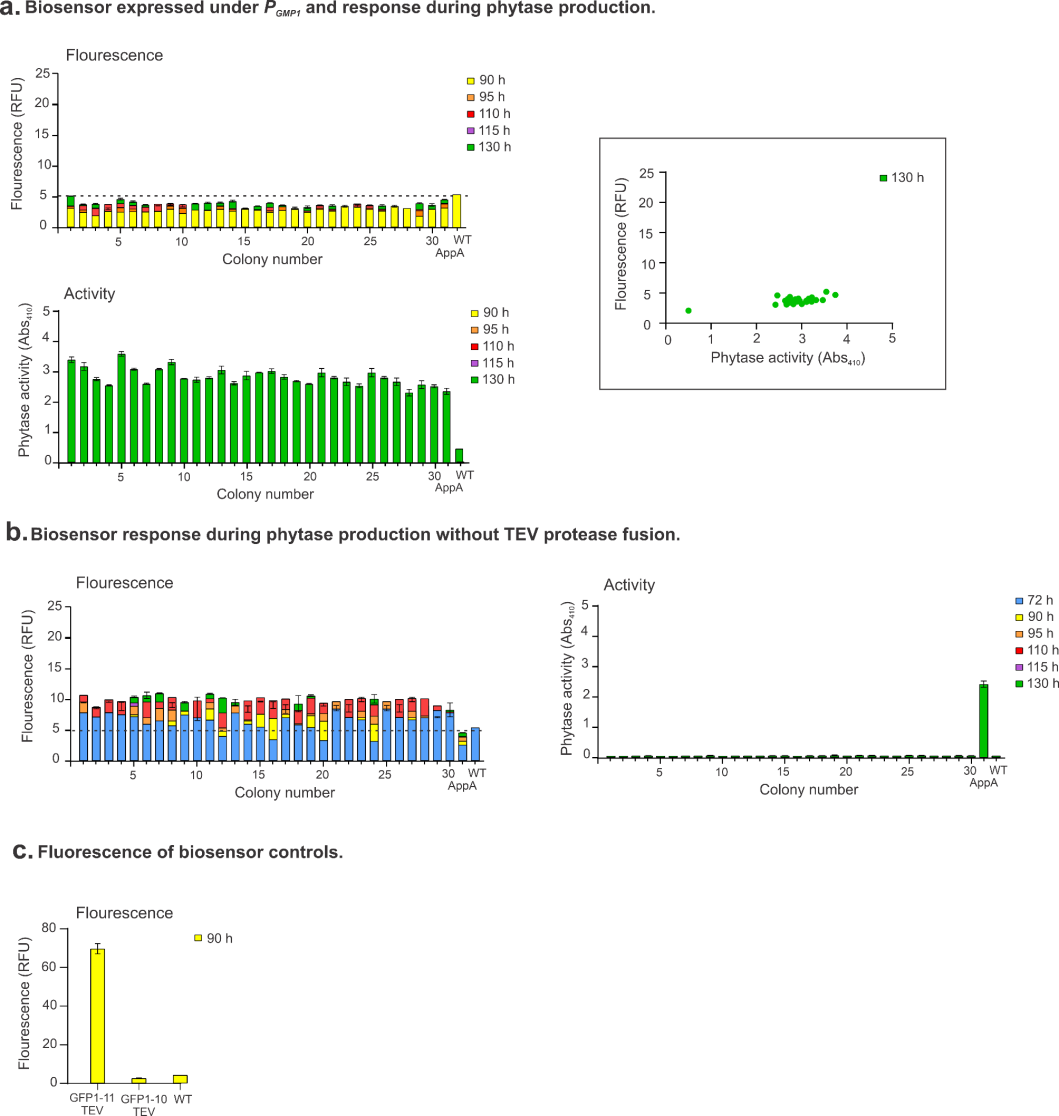
**Biosensor response during phytase production. a**, Biosensor expressed under *P*_*GMPI*_ and response during phytase production. **b**, Biosensor response during phytase production without the TEV protease fusion. Phytase production under methanol induction at 72, 90, 95, 110, 115 and 130 h. Fluorescence is indicated as relative fluorescence units (RFU) at 485–520 nm and phytase activity as absorbance at 405 nm. Correlation between secreted enzyme activity and intracellular GFP fluorescence shown in framed graph. Data are shown as the mean and standard deviation of three biological replicates. The dashed line indicates background fluorescence at 485–520 nm. **c**, Fluorescence of the biosensor controls. Complete biosensor GFP1-11_TEV protease (GFP1-11_TEV), biosensor fragment GFP1-10_TEV protease (GFP1-10_TEV) and wild type MutS strain (WT).

The GFP1-10_TEV protease fusion expressed under the *P*_*GPM1*_ promoter did not appear to generate sufficient quantities of protein to measure an increase of fluorescence during expression of tagged AppA phytase (Fig. 4a). Phytase activity assays showed secretion of phytase confirming the expression of phytase enzyme in these strains but no increase fluorescence from the background was observed (Fig. 4a).

We also constructed a second biosensor design without the inclusion of TEV protease which also works as a control for protease activity (Fig. 3d). A consideration with this design is that after folding of complete GFP, the recombinant protein could be retained intracellularly in the ER due to attachment to GFP, which decreases the amount of secreted recombinant protein. Alternatively, the complete GFP could be secreted through attachment to the recombinant protein, obtaining a fusion of GFP-recombinant protein in the supernatant.

The biosensor design without the TEV protease fusion, GFP1-10 alone, showed very low secretion of phytase for all transformants screened (Fig. 4b). This result suggests the enzyme is most likely retained in the ER. No fluorescence in the supernatant was observed due to secretion of complete GFP to the media (data not shown). Additionally, this biosensor design showed lower fluorescence intensity compared to the GFP1-10_TEV protease fusion. We speculate that the attachment of the recombinant protein, AppA, creates a potential steric hindrance for complete GFP folding since no cleavage can occur due to absence of TEV protease.

Control strains expressing complete GFP fused to TEV protease showed higher fluorescence intensity than strains expressing the split fragments. This observation is most likely due to the lower amount of recombinant GFP11 fusion protein expressed, limiting the total amount of folded complete GFP. Since the GFP11 tag is cleaved by TEV protease the recombinant protein should not create steric hindrance on GFP folding. Fluorescence intensities from biosensor controls are shown in Fig. 4c.

### Flask fermentation of phytase and laccase production transformants confirms biosensor-guided screening

From the screening of phytase and laccase producers using biosensor fluorescence response, we selected six strains for scale up production in flasks and confirmation of the screening result. In the case of phytase, the biosensor indicated colony numbers 9 and 17 to be the best enzyme producers from the thirty screened, which corresponded to the phytase assay. We grew these colonies in 50 mL cultures together with colony number 2 as a mid-low producer control and induced with methanol additions until harvest at 130 h. Protein concentration was determined using the Bradford assay [4]. Results confirmed colonies 9 and 17 secreted high amounts of phytase to the extracellular media, 175 and 215 µg/mL, respectively, while colony number 2 presented very lower production at 32 µg/mL.

For laccase transformants, we selected colony numbers 9 and 13 as high producers and 1 and 11 as low producers (Fig. 2). Protein concentration in the supernatant was very low for all colonies analysed, and SDS-PAGE analysis (data not shown) confirmed that although colonies 9 and 13 were intermediate laccase producers, low total protein was detected in the supernatant at 40 and 69 µg/mL, respectively, while no laccase in the supernatant could be quantified for colonies 1 and 11 as expected from the initial screen. Since the biosensor showed some relative fluorescence for colonies 1 and 11 (Fig. 2b), it was possible that there was some production of intracellular laccase that failed to be secreted. Enzymatic assay using intracellular extract did not show laccase activity (data not shown). Overall, the results obtained from phytase and laccase production confirmed the biosensor can identify superior protein production clones.

**Table 1:** **Biosensor design and strain names used in this work.** The bidirectional promoters used for expression of heterologous proteins, phytase, laccase, β-casein and β-lactoglobulin fused to GFP11 fragment and the TEV protease fusions to GFP1-10 fragment are indicated.

Strain name	Promoter	GFP	TEV-P	Recombinant protein fusion.
GFP_AppA	*P*_*CAT1−PGAP*_	GFP 1–10	+	AppA phytase_GFP11.
GFP_TEV_AppA	*P*_*CAT1−PGAP*_	GFP 1–10	+	AppA phytase_GFP11.
GFP_*PGM1*__TEV_AppA	*P*_*CAT1−PGPM1*_	GFP 1–10	+	AppA phytase_GFP11.
GFP1-11_TEV	*P*_*CAT1−PGAP*_	GFP 1–11	+	-
GFP1-10_TEV	*P*_*GAP*_	GFP 1–10	+	-
GFP_TEV_Thi	*P*_*GAP*_	GFP 1–10	+	Thi laccase_GFP11.
GFP_TEV_ β-cas	*P*_*HpFMD−PGAP*_	GFP 1–10	+	β-casein_GFP11.
GFP_TEV_ β-lac	*P*_*CAT1−PGAP*_	GFP 1–10	+	β-lactoglobulin_GFP11.
AppA	*P*_*CAT1*_	-	-	AppA phytase.
Thi	*P*_*HpFMD*_	-	-	Thi laccase.

### Expression of structural proteins β-lactoglobulin and β-casein and determination of biosensor fluorescence

Following validation of the biosensor-guided screening for enzyme expression, we investigated if the biosensor could be used for the screening of strains expressing structural proteins. Screening of structural proteins represents a challenge due to the difficulty of analytical methods for determination of relative protein amounts. Two good examples of relevant importance for the food industry are β-lactoglobulin and β-casein from bovine milk. We selected bovine β-lactoglobulin and β-casein proteins and fused them to the GFP11 tag together with a TEV cleavage site as previously described (Fig. 1). While β-lactoglobulin is a whey soluble protein in animal milk, β-casein is intrinsically difficult to quantify due to its insolubility [19, 20]. To make sure we had enough amount of β-lactoglobulin and β-casein proteins expressed for the sensitivity of protein quantifications methods, we changed the *P*_*CAT1*_ promoter in our previous designs for the stronger de-repressed methanol inducible promoter *P*_*HpFMD*_ [21]. We determined β-lactoglobulin concentration in the supernatant by the Bradford assay.

**Fig. 5:**
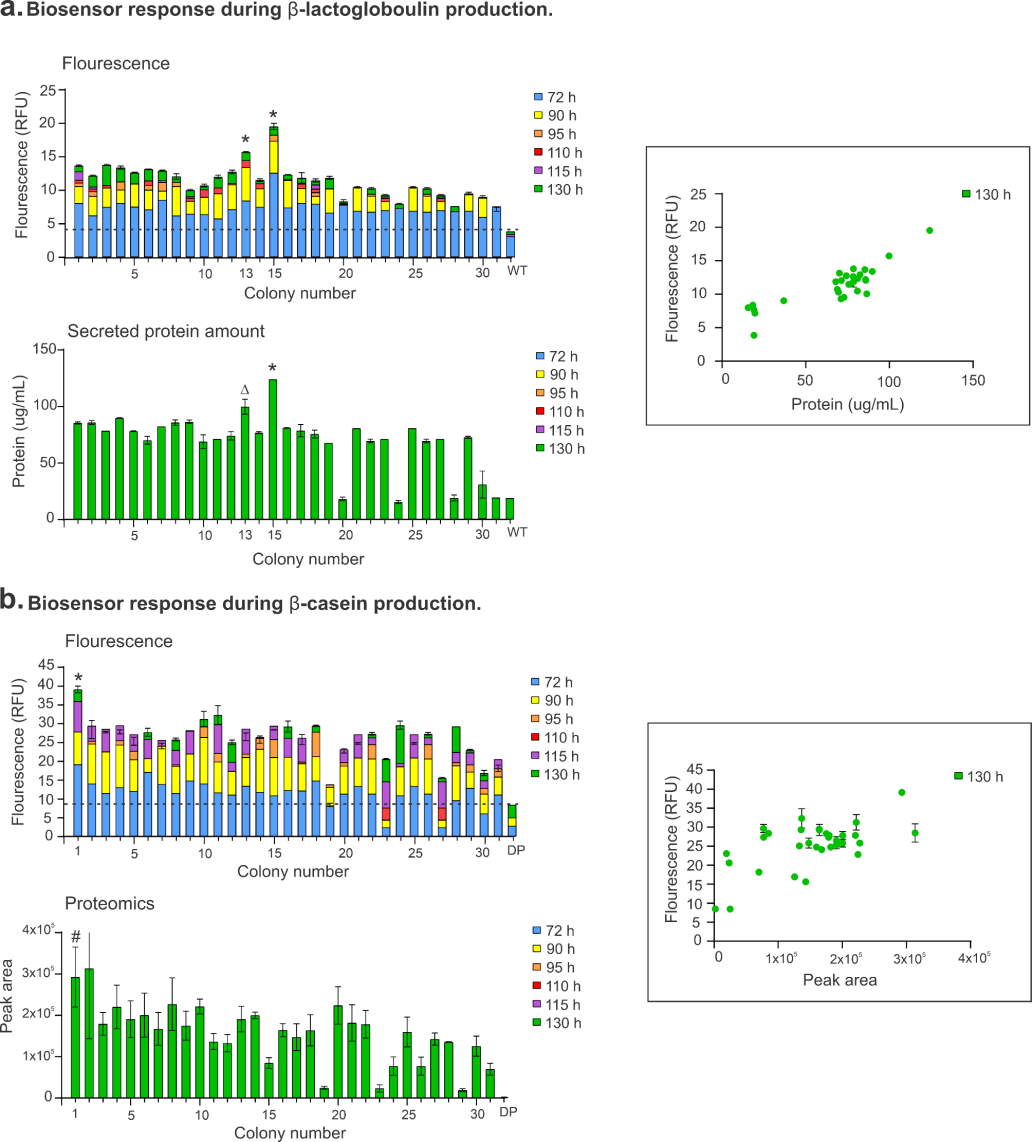
**Biosensor response during β-lactoglobulin and β-casein production. a**, β-lactoglobulin and **b**, β-casein under methanol induction at 72, 90, 95, 110, 115 and 130 h. Fluorescence is indicated as relative fluorescence units (RFU) β-lactoglobulin and β-casein abundance by protein concentration and proteomics analysis in the supernatant, respectively. Correlation between secreted protein abundance and intracellular GFP fluorescence shown in framed graphs. Data are presented as mean and standard deviation of biological replicates and analysed using one-way ANOVA with multiple comparisons. Dash line indicates background fluorescence at 485–520 nm. *Significantly different to all colonies at 130 h (p < 0.01). ^#^ Significantly different to colonies 15, 19, 23, 24, 26, 29, 31, 32 and protease deficient strain (DP) at 130 h (p < 0.01). ^Δ^ Significantly different to all colonies except 1, 2, 4, 8 and 9 at 130 h (p < 0.01)

Fluorescence intensity increases during production of β-lactoglobulin following methanol induction confirmed the biosensor can be used for screening of difficult structural proteins (Fig. 5a). Colony numbers 13 and 15 showed best production of β-lactoglobulin by total protein amount in the supernatant, which corresponded to an increase of fluorescence at 485–520 nm. On the other hand, colony numbers 20, 24 and 30 showed no production of β-lactoglobulin by protein quantification and no fluorescence increase at 485–520 nm during methanol induction (Fig. 5a).

Due to the insolubility of β-casein and the associated challenge of β-casein quantification by standard methods like the Bradford assay, we employed a bottom-up proteomics approach whereby three β-casein peptides generated upon trypsin digestion were monitored quantitatively by liquid chromatography-multiple reaction monitoring-mass spectrometry (LC-MRM-MS). Furthermore, we used a protease deficient strain (*pep4*Δ, *prb1*Δ) (BG16, ATUM) as the parental strain for production of β-casein. Previous results conducted on a non-protease deficient strain (BG11, ATUM) showed an abundance of semi-tryptic and non-tryptic peptides during analysis on a high-resolution LC-MS platform, suggesting potential degradation of β-casein (data not shown).

An increase in fluorescence during methanol induction confirmed the biosensor can be used for selection of insoluble protein producers. Figure 5b indicates colony 1 is a good β-casein producer from fluorescence intensity as well as from proteomics analysis by LC-MRM-MS. On the other hand, colonies 19 and 23 showed low fluorescence intensity which was in agreement with the low abundance of casein peptides measured by MS.

We also determined the cell density (OD_600_) of the β-casein producing strains. We observed variation in OD_600_ between different strains, however, since β-casein is an insoluble protein with tendency to form aggregates, OD_600_ measurement might be affected by light scattering caused by these protein aggregates (Additional file 1 Fig. 1).

## Discussion

Here we developed a biosensor for screening of production clones in *P. pastoris* using fluorescence. We designed the biosensor based on a split-GFP system and fused it to a TEV protease to facilitate secretion of target proteins. Four recombinant proteins were tested to validate the biosensor system: the enzymes phytase and laccase, and structural proteins β-casein and β-lactoglobulin. The results suggest the split-GFP biosensor could be applied to screen any secreted recombinant protein to enable selection of high producer clones in industrial settings.

In this study we focused on the screening of production clones of two industrially relevant enzymes, as well as two structural proteins, one of which is inherently challenging to quantify by standard analytical methods. However, the biosensor could be used for screening of any secreted recombinant protein of interest if the split-GFP small fragment is fused to the C-terminal region. The incorporation of TEV fusion to the large GFP fragment allows to secretion of untagged recombinant protein to the extracellular media.

We showed that the fluorometric response of the biosensor correlates with the amount of secreted protein by enzymatic assay determination for phytase and laccase, total protein quantification for β-lactoglobulin and proteomics assay for β-casein. In industrial settings, the biosensor can measure recombinant proteins without accumulating product in the extracellular medium for quantification.

Many biological signalling systems have been assembled for a variety of applications including molecular diagnostics, cell-based biosensors, therapeutics and industrial biotechnology [22]. Unlike transcription-based sensors, where reporter genes are placed under the control of DNA regulating elements to follow signalling cascades, the split-GFP biosensor design works through direct folding of an already synthesised large GFP fragment. Protein based biosensors have the advantage of directly responding faster to due to protein-protein interactions and have not been previously reported as a screening tool for production clones in *P. pastoris* [22]. Maturation of complete GFP did not appear to affect the functionality of the biosensor for screening processes, with fluorescence intensity increasing during methanol induction.

Recently a flavin-based fluorescent protein reporter, iLOV, has been developed for screening *P. pastoris* strains for production of antimicrobial peptide epidermicin NI01 [23]. This system is based on a secreted fusion of iLOV_NI01 which requires additional protease cleavage to release the NI01 peptide. Our split-GFP_TEV protease biosensor is intracellularly expressed and does not require any further processing outside the cell which facilitates downstream processes in industrial settings.

It could have been possible that the expression of the biosensor could affect the growth rate of production strains which is undesirable in industrial settings. The co-expression of the biosensor together with the recombinant protein of interest could create a burden in the protein production machinery decreasing strain overall productivity. We did not observe a decrease in the production of recombinant proteins using the biosensor design or decrease in growth rate. In the case of phytase, production titres of selected transformants were comparable to a previously selected high titre phytase production strain expressed under the control of the same *P*_*CAT1*_ promoter without biosensor integration [15]. Nevertheless, for industrial applications, the biosensor could be flanked by cre/lox sequences for removal using a recombinase system after screening and selection of clones [24]. This approach is commonly used to eliminate antibiotic markers in selected strains for certain industrial applications including food grade products.

## Conclusions

The split-GFP biosensor effectively selects for high production clones of secreted recombinant proteins in *P. pastoris*. Here, we screened for high producer clones for industrial enzymes phytase and laccase and structural milk proteins β-casein and β-lactoglobulin by fluorescence intensity during methanol induction. Quantification of proteins in the extracellular media was used to confirm correct selection of strains using the biosensor design. Finally, flask fermentations were carried out to produce recombinant proteins from high and low production clones to confirm sensor utility. Overall, the results show that the biosensor can be used to accelerate the efficient screening of *P. pastoris* clones for recombinant protein secretion, providing the opportunity for higher-throughput screening and the identification of industrially useful strains.

## Materials and methods

### Strains and growth conditions

The strains used in this study were the *P. pastoris* BG11 strain (derivative of *P. pastoris* BG10 strain, *Δaox1*) and BG16 (*pep4*Δ, *prb1Δ*, mut+) strain from ATUM Inc. (Newark, California, USA). NEB® 5-Alpha Competent *E. coli* (NEB, Australia) was used for cloning. Cultivations were conducted in Luria Broth (LB) media for *E. coli* and yeast cultures were either grown in YPD medium (1% w/v yeast extract, 2% w/v peptone and 2% w/v glucose), buffered minimal dextrose (BMD) medium (1.34% Yeast Nitrogen Base YNB, 4 × 10^− 5^% biotin, 200 mM potassium phosphate buffer pH 6.0 and 2% glucose), buffered minimal methanol (BMM) medium (1.34% YNB, 4 × 10^− 5^% biotin, 200 mM potassium phosphate buffer pH 6.0) with 1% methanol (BMM2) or 5% methanol (BMM10). Antibiotic Zeocin (Invitrogen) was added to the media when required at a final concentration of 25 µg/mL for *E. coli* or 200 µg/mL for *P. pastoris* cultivations.

### Cloning and transformation of ***P. pastoris***

Split-GFP1-10 large fragment sequence was codon optimised and fused to codon optimised TEV protease from *Tobacco Etch Virus* at the C-terminal end gene sequences [12]. Linker sequence SGGS was included between the GFP1-10 sequence and TEV protease sequence. Fused genes were synthesised and cloned by GeneWiz (Azenta Life Sciences) into pD912 derived vector (ATUM Inc.) with protein disulfide isomerase signal peptide for ER transporting and a HDEL sequence was added to the C-terminal sequence to maintain the fusion in the lumen of the ER [25]. Protein sequences from *E. coli* AppA phytase, *Tramates hirsuta* laccase, bovine β-casein and bovine β-lactoglobulin were codon optimised then synthesised and cloned into the same pD912 derived vector containing GFP10-TEV fusion by GeneWiz (Azenta Life Sciences). The α-mating-factor secretion signal added at the N-terminus for targeting secretion and split GFP11 small fragment added to the C-terminus together with TEV protease cleavage site. The glyceraldehydes-3-phosphate dehydrogenase constitutive promoter (*P*_*GAP*_) was used for expression of GFP1-10_TEV protease fusion and the catalase 1 promoter (*P*_*CAT1*_) was used for expression of recombinant protein_GFP11 fusion. Biosensor control sequences were also cloned into pD912 derived vector transformed in *P. pastoris* for control experiments (construct and plasmids sequences can be found in Additional file 2). All plasmids where linearised with *Swa*I restriction enzyme and used to transform *P. pastoris* BG11 according to protocols by Navone et al. [15]. After the transformation, screening of transformants was performed in DWP as described previously [26].

### Protein expression in ***P. pastoris***

Recombinant protein expression was conducted in 250 mL baffed shake flasks following standard expression conditions at 28 °C, 250 rpm. The culture was grown in 50 mL of BMD1 for 65 h following methanol induction with BMM10 and consecutive additions of pure methanol 1% final concentration until harvest at 130 h. Protein concentration in the culture supernatant was determined using the Bradford method [4].

### Green fluorescence measurements

Total cell fluorescence was measured at 480–520 nm in a Varioskan Lux microplate reader. Briefly, 20 µL of cells were taken from 96-deep-well screening plates at 72, 90, 95, 110, 115 and 130 h and resuspended in 180 µL of water immediately prior to measuring.

### Enzymatic assays

#### Phytase

Acid phosphatase activity was assayed to measure phytase expression levels using a assay based on the substrate *para*-nitrophenylphosphate (*p*-NPP) (Sigma) at an initial concentration of 5 mM in the assay [27]. Briefly, 10 µL of *P. pastoris* supernatant from 96-deep-well plates was incubated with 90 µL of p-NPP substrate in 250 mM sodium acetate buffer pH 5.5 for 15 min at 37 °C. The reaction was stopped by the addition of 10 µL of 1 M NaOH. The released *para*-nitrophenol was measured at 410 nm after 10 min incubation at room temperature. Reactions were conducted in triplicate in 96-well plates.

#### Laccase

Supernatants were harvested and copper chloride was added to a final concentration of 100 µM. The substrate, 2,2’-azino-bis(3-ethylbenzothiazoline-6-sulfonic acid) (ABTS), was added to supernatant samples at a final concentration of 4 mM in a 96-well plate (180 µL supernatant plus 20 µL of 40 mM ABTS). The change in absorbance at 405 nm corresponding to oxidation of ABTS was monitored at 30 °C.

### Proteomics assay

The content of each well (~ 200 µL) was mixed with solubilisation buffer (8 M urea, 10% ACN, 100 mM Tris pH 8.0) at 1:2 ratio (v/v) and agitated for 1 h at RT. Samples were diluted 6 times using dilution buffer (10% ACN, 100 mM Tris pH 8.0), spiked with 2 µL trypsin working solution (0.1 µg trypsin [Promega], 10% ACN, 100 mM Tris pH 8.0) and agitated overnight at 37 °C. Next day, all digests were mixed with 4% TFA at 1:1 ratio (v/v) and centrifuged at 18,000 xg for 15 min at 21 °C. An equal aliquot (1 mL) of each supernatant was then desalted using StageTips filled with SCX membrane following procedure described previously [28]. After drying in vacuum concentrator without heating, peptide samples were reconstituted in 0.1% FA and 10 µL was analysed by liquid chromatography-multiple reaction monitoring-mass spectrometry (LC-MRM-MS).

LC-MRM-MS was conducted using Shimadzu LCMS-8050 instrument equipped with DUIS ion source and coupled to a Nexera UPLC system (Shimadzu). Reversed-phase chromatography separation was performed on a Kinetex 2.6 μm EVO C18 100 A, 100 × 2.1 mm column (Phenomenex) at flow rate of 0.5 mL/min and using 0.1% FA in water as mobile phase A and 0.1% FA in ACN as mobile phase B. Peptides were separated using a linear gradient of mobile phase B as follows: 0 to 0.51 min 2% B, 0.51 to 3 min 2–35% B, 3.01 to 3.50 min 85% B, 3.51 to 5 min 2% B. Data acquisition parameters can be found in Additional file 1. Relative quantitation was conducted from the areas under chromatographic peaks in Skyline software.

### Statistical analysis

All data are presented as mean ± SD for each group and analysed by ANOVA using GraphPad Prism. A p-value of less than 0.01 or 0.001 was considered statistically significant.

## Electronic supplementary material

Below is the link to the electronic supplementary material.

**Additional file 1: Fig. 1.** Optical density of biosensor expressing strains. Absorbance at 600 nm of biosensor strains expressing phytase, laccase, β-casein and β-lactoglobulin. **Table 1.** Data aquation parameters for liquid chromatography-multiple reaction monitoring-mass spectrometry (LC-MRM-MS).

**Additional file 2:** DNA sequences for biosensor design and regulatory elements.

## Data Availability

All datasets generated for this study are included in the manuscript and Additional files.
