# Supplementary material for: Biosensor-guided rapid screening for improved recombinant protein secretion in Pichia pastoris
Source: Microb Cell Fact. 2023 May 3;22:92. doi: 10.1186/s12934-023-02089-z (PMC10155391; doi:10.1186/s12934-023-02089-z)
Supplement: Supplementary file 2 — Additional file 2: DNA sequences for biosensor design and regulatory elements. [file 12934_2023_2089_MOESM2_ESM.docx]

**Additional Information for ‘Biosensor-guided rapid screening for improved recombinant protein secretion in *Pichia pastoris*’.**

Laura Navone^1,2^, Kaylee Moffitt^1^, James Behrendorff^1,2^, Pawel Sadowski^3^, Carol Hartley^4^ and Robert Speight^1,2^.

^1^ School of Biology and Environmental Sciences, Faculty of Science, Queensland University of Technology (QUT), Brisbane, Queensland, 4000, Australia.

^2^ ARC Centre of Excellence in Synthetic Biology, Queensland University of Technology (QUT), Brisbane, Queensland, 4000, Australia.

^3^ Central Analytical Research Facility (CARF), Queensland University of Technology (QUT), Brisbane, Queensland, 4000, Australia.

^4^ CSIRO Environment, Canberra, ACT 2600, Australia.

*Corresponding author: [laura.navone@qut.edu.au](mailto:laura.navone@qut.edu.au), Queensland University of Technology, Brisbane, Queensland, Australia.

**
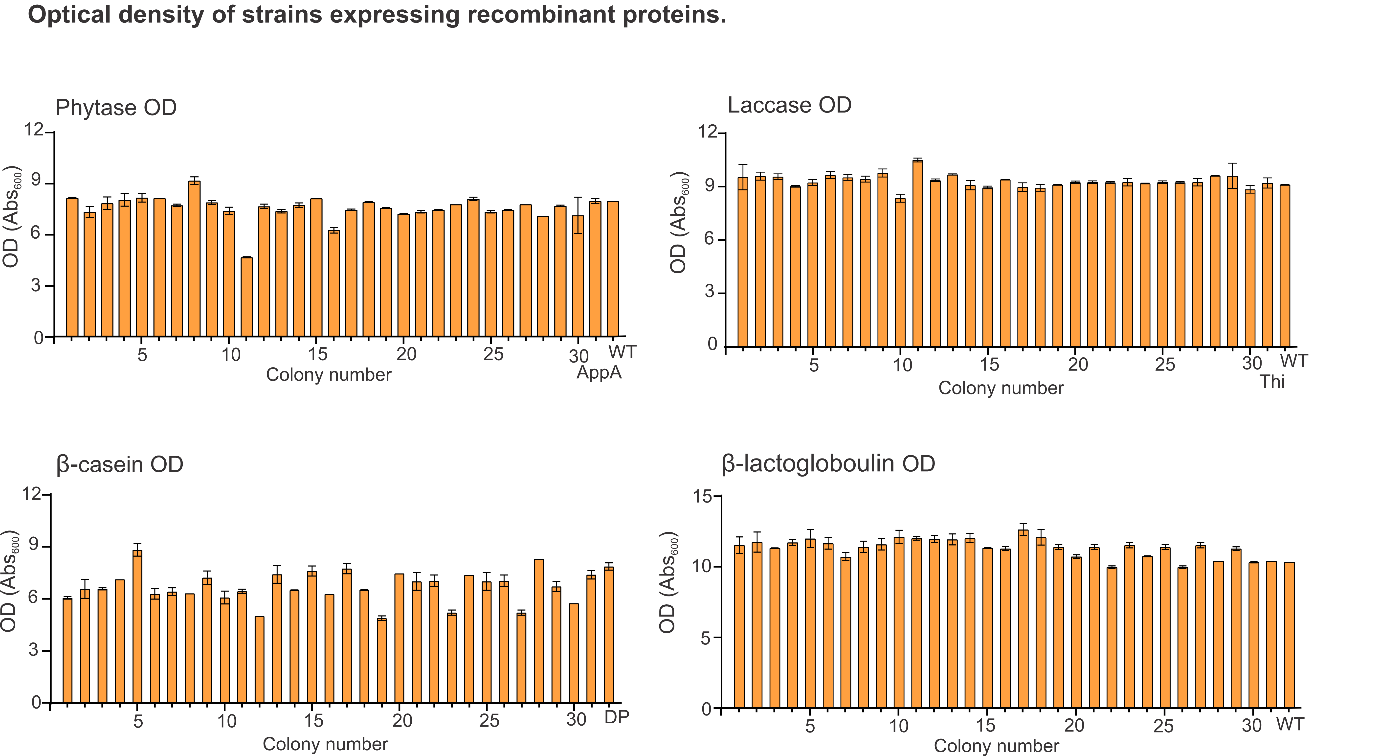
**

**Supplementary Fig. 1.** Optical density of biosensor expressing strains. Absorbance at 600 nm of biosensor strains expressing phytase, laccase, β-casein and β-lactoglobulin.

**
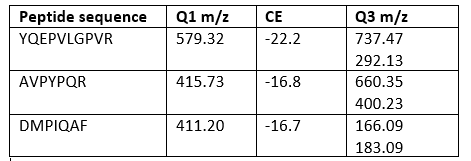
**

**Supplementary Table 1.** Data aquation parameters for liquid chromatography-multiple reaction monitoring-mass spectrometry (LC-MRM-MS).
